# A Case of Radical Cure of Hydrocele

**Published:** 1866-06

**Authors:** H. Rogers

**Affiliations:** Dunkirk, N. Y.


### A Case of Radical Cure of Hydrocele.

BY H. ROGERS, M. D., DUNKIRK, N. Y.

*To the Editor of the Buffalo Medical and Surgical Journal:*

Dear Doctor:—The following case of radical cure of hydrocele, recently reported at a meeting of the Lake Shore Medical Society, may not be uninteresting to your readers. I report it with the hope that further experiments may be made to test the value of the new remedy employed. The previous history of the case will add to its interest and value, and I will give it and my treatment, as concisely as possible:

J. T., a young man about thirty years of age, of full habit and good constitution, first noticed the approach of his malady about five years since. Its progress was slow and was treated by suspensory bandage and topical applications. Later, injections of iodine were used, and injections of port wine, and, failing in these, he consulted a distinguished surgeon of Cleveland, who operated by making a free incision through the scrotal sac two inches in length, with no better results. Despairing of permanent benefits from severe operations he applied to me in 1864, on several occasions, for temporary relief, which was afforded him. In June, 1865, I was permitted to make an effort to effect a permanent cure. I injected the sac with a solution of ammonia ferric alum, in the proportion of ten grains to the ounce of water, retaining it for several minutes. No pain or inconvenience followed this application, and contrary to advice, he used no other precaution to prevent accident than to wear a suspensory bandage for a few days. He continued in the discharge of his usual duties. Thus the result was a cure, painless, immediate and permanent.

I would remark that in this instance I prepared the patient for the use of the ammonia ferric alum as I am accustomed to prepare them for the use of iodine, etc., and which I regard as an important element of success in operations for radical cure by injections, by emptying the sac, if large, several days previously to introducing the injection, thus giving the distended parts time to contract well, in which condition the remedial agent acts with more efficiency.

I was led to apply this agent in this instance from the success attending my use of it in hemorrhages of various kinds, and in leucorrhœa, gleet, etc., it having a marked tonic and astringent effect. Other cases of the uses of this remedy may not be so successful, yet it is to be desired, that instances may be multiplied and the results reported.

